# Assumptions Matter: The Long-Term Cost Analysis of IsaVRd vs DVRd

**DOI:** 10.36469/001c.145075

**Published:** 2025-10-17

**Authors:** Feng Lin, Audrey Petitjean, Medha Sasane

**Affiliations:** 1 Sanofi, Morristown, New Jersey, USA; 2 Sanofi, Lyon, France; 3 Sanofi, Cambridge, Massachusetts, USA

**Keywords:** isatuximab, daratumumab, cost comparison, long-term economics, newly diagnosed multiple myeloma

We reviewed the article by Gupta-Werner et al^1^ with great interest. This analysis compared the drug acquisition costs (DAC) of the combination of bortezomib, lenalidomide, and dexamethasone with daratumumab (DVRd) vs isatuximab (IsaVRd) in the first 2 years of frontline treatment for patients with transplant-ineligible newly diagnosed multiple myeloma (NDMM) in the United States. While we acknowledge the authors’ effort, several assumptions in the methodology raise concerns about the validity and generalizability of their conclusions. These assumptions include patient weight and dose rounding practices, the omission of relative dose intensity (RDI), and the use of a limited time horizon, which may not fully reflect real-world clinical practices.

## Patient Weight and Dose Rounding

The analysis assumed a median patient weight of 82 to 85 kg,^1^ which is approximately 15% higher than the 72.2 kg median weight reported in the IMROZ trial.^2^ Furthermore, the authors used a dose rounding-up practice for isatuximab cost calculation, rounding from 820 mg to 900 mg.^1^ While dosing rounding is a common practice in oncology, guidelines from organizations like the Hematology/Oncology Pharmacy Association (HOPA) suggest a rounding margin of within ±10% to balance efficacy and toxicity,^3^ with some institutions recommending an even tighter threshold (±5%). HOPA also suggests that rounding down is a safer approach for monoclonal antibodies to mitigate the risk of dose-limiting toxicities without compromising efficacy.^3^ By rounding the dose up, the analysis by Gupta-Werner et al may have introduced a bias in favor of the fixed-dose subcutaneous formulation of daratumumab, potentially overstating the cost of isatuximab.

A more accurate analysis using the 72.2 kg median weight reported in the IMROZ trial would expect a full prescribed isatuximab dose of 722 mg. Rounding the dose down to 700 mg represents a 3% reduction, which is well within HOPA’s recommended threshold and aligns with the goal of minimizing drug waste while prioritizing patient safety. Rounding the dose up to 800 mg, however, represent a 10.8% increase, which exceeds the HOPA recommended threshold and contributes to a biased calculation of isatuximab cost. Even using a higher median weight of 82 kg, rounding the isatuximab dose down from 820 mg to 800 mg represents a 2.4% reduction, which is well within the HOPA threshold. Conversely, rounding the dose up to 900 mg (9.8% increase) nearly exceeds the recommended threshold.

To account for variability in weight, we provide treatment costs across 3 weight ranges: 66-75 kg, 76-85 kg, and 86-95 kg. For a patient weighting between 76 and 85 kg, the required isatuximab dose is between 760 and 850 mg. Following the HOPA guidance, a dose rounded within ±10% margin would require one 500 mg vial and three 100 mg vials of isatuximab. This results in a DAC of $5901 per administration of isatuximab, which is 22% lower than the DAC estimated in Gupta-Werner et al.^1^ Because daratumumab SC is fixed dose, the DAC of DVRd does not vary by patient weight. Our updated calculation showed that isatuximab’s DAC was $12 036 lower in Year 1, $9300 lower in Year 2, and $49 764 lower in Year 3 and beyond than daratumumab (**Supplementary Table S1** and **Figure S1a**).

## Limited 2-Year Time Horizon

A major limitation of the cost analysis by Gupta-Werner et al is the 2-year time horizon.^1^ The 2-year duration is insufficient to capture the full economic impact of prolonged frontline treatment for NDMM. The median treatment durations was 56.3 months for DVRd^4^ and 55.3 months for IsaVRd,^2^ making a longer-term analysis essential for a transparent and robust cost comparison.

Although the costs of the two regimens appear similar in the first 2 years, the cumulative cost of IsaVRd becomes lower than that of DVRd starting in Year 3 (**Supplementary Figure S1b**). This is because the dosing frequency of isatuximab decreases from week 77 onward, matching the 13 administrations per year for daratumumab (**Supplementary Table S1**).

Over a 5-year period, the cumulative treatment cost of the IsaVRd regimen was consistently lower than that of the DVRd regimen across all patient weight ranges (**Supplementary Figure S1b**). IsaVRd incurs higher costs in the first 2 years, but only for patients weighing over 86 kg. The 2-year analysis by Gupta-Werner et al^1^ significantly misrepresents the long-term economic profiles of these two regimens, which is a critical consideration given the goal of sustained treatment for NDMM.

## Omission of Relative Dose Intensity

The Gupta-Werner et al analysis overestimated the DAC for both regimens because it did not account for relative dose intensity (RDI). In clinical practice, factors like comorbidities, poor tolerability, and drug toxicities often lead to dose reductions or delays, preventing cancer patients from receiving the full prescribed dose. This directly affects the actual amount of drug administered. As the HOPA guideline advised against upward dose rounding for patients with major organ dysfunction or poor performance status, the neglect of RDI can significantly misrepresent the true DAC.

To address this limitation, we incorporated RDI data from clinical trials in our analysis. The IMROZ trial reported the median RDI for both IsaVRd and VRd regimens. Specifically, the median RDI was 93.6% for isatuximab, 90.3% for bortezomib, 77.7% for lenalidomide, and 81.6% for dexamethasone for the IsaVRd arm.^2^ For the VRd arm, the median RDI was 86.7% for bortezomib, 83.5% for lenalidomide, and 79.3% for dexamethasone.^2^ Although the median RDI for DVRd was not explicitly reported, it’s reasonable to infer its RDI from other daratumumab trials in transplant-ineligible NDMM, such as MAIA (98.4%) and ALCYONE (99.3%). We also assumed the RDI of its VRd component would resemble the RDI reported in the IMROZ trial.

Incorporating these RDI values in our calculation for patients weighing 76-85 kg, the 5-year cumulative treatment cost for IsaVRd showed a 17% savings and DVRd showed a 14% savings.

## Additional Consideration: 30-Minute Infusion of Isatuximab

While the standard isatuximab infusion is 75 minutes, a shorter 30-minute infusion has been shown to be a feasible, well-tolerated, and more convenient administration alternative in the Phase 1b TCD13983 study.^5^ This rapid infusion method has since been adopted in clinical practices, reducing patient time in the clinics and improving overall patient experience.^5^ The adoption of this alternative positions the 30-minute isatuximab infusion as a compelling economic alternative to daratumumab SC. By improving efficiency and reducing the operational burden on the healthcare system, it positively impacts the total cost of care.

To reflect the economic impact of this alternative, we analyzed the administration costs for daratumumab SC, standard isatuximab IV infusion, and the 30-minute isatuximab infusion from week 7 onward. The 30-minute infusion reduced drug administration cost by 42% in the first year and by 52% in year 2 and beyond compared to the standard isatuximab infusion (**Supplementary Figure S1d**). This rapid infusion method would therefore further improve the economic advantage of the IsaVRd regimen, which has already demonstrated a cost saving over DVRd.

## Conclusions

The analysis by Gupta-Werner et al, which favors DVRd, is driven by methodological limitations. Their analysis relies on biased assumptions including an inflated median patient weight, improper dose rounding, the abbreviated time horizon and the omission of RDI. When these critical assumptions were properly adjusted, IsaVRd emerged as the more economically advantageous frontline treatment for patients with transplant-ineligible NDMM. This finding is further strengthened by the clinical availability of a 30-minute isatuximab infusion, which offers a key advantage by improving both patient care and economic efficiency. Ultimately, a comprehensive analysis that accounts for these factors is essential for providing a transparent and accurate economic profile of these vital treatment options.

Feng Lin

Sanofi, Morristown, New Jersey, USA

Audrey Petitjean

Sanofi Lyon, France

Medha Sasane

Sanofi, Cambridge, Massachusetts, USA

### Disclosures

F.L., A.P., and M.S. are employees of Sanofi and may own shares/stock options of Sanofi.

## Supplementary Material

Online Supplementary Material

## Data Availability

Data sharing is not applicable to this article as no datasets were generated or analyzed for this publication.
