# Supplementary material for: Assumptions Matter: The Long-Term Cost Analysis of IsaVRd vs DVRd
Source: J Health Econ Outcomes Res. 2025 Oct 17;12(2):145–6. doi: 10.36469/001c.145075 (PMC12535738; doi:10.36469/001c.145075)
Supplement: Online Supplementary Material [file jheor_2025_12_2_145075_306599.pdf]

Journal of Health Economics and Outcomes Research

Online Supplementary Material

Assumptions Matter: The Long-Term Analysis of IsaVRd vs DVRd. *JHEOR*. 2025;12(2):145-146. doi:10.36469/jheor.2025.145075

Table S1: Number of Doses, Drug Acquisition Cost, and Administration Cost of DVRd and IsaVRd Across Weight Ranges 66-75 kg, 76-85 kg, and 86-95 kg

Figure S1: Effect of Variable Assumptions on a Comprehensive Economic Analysis on Drug Acquisition Cost, Administration Cost, and Total Treatment Cost of DVRd and IsaVRd

This supplementary material has been provided by the authors to give readers additional information about their work.

This is an open-access article distributed under the terms of the Creative Commons Attribution 4.0 International License (CCBY-4.0). View this license's legal deed at http://creativecommons.org/licenses/by/4.0 and legal code at http://creativecommons.org/licenses/by/4.0/legalcode for more information.

Table 1. Number of Doses, DAC, and Administration Cost of DVRd and IsaVRd Across Weight Ranges 66-75 kg, 76-85 kg, and 86-95 kg

	DVRd					IsaVRd				
	**Year 1**	**Year 2**	**Year 3**	**Year 4**	**Year 5**	**Year 1**	**Year 2**	**Year 3**	**Year 4**	**Year 5**
No. of doses										
Anti-CD38[a]	19	13	13	13	13	28	19	13	13	13
Bortezomib SC	32	0	0	0	0	32	0	0	0	0
Lenalidomide	259	273	273	273	273	259	273	273	273	273
Dexamethasone	92	52	52	52	52	96	52	52	52	52
Cost of drug administration,[b,c] $										
Anti-CD38[a]	1330	910	910	910	910	6264	4174	2856	2856	2856
Bortezomib SC	2240	0	0	0	0	2240	0	0	0	0
Total administration cost	3570	910	910	910	910	8504	4174	2856	2856	2856
**Scenario 1: Median weight 76-85 kg, HOPA rounding guidance applied (one 500 mg vial and 3-100 mg vials of isatuximab)**										
DAC of each drug per dosing schedule, $										
Anti-CD38[a]	200 868	137 436	137 436	137 436	137 436	188 832	128 136	87 672	87 672	87 672
Bortezomib SC	1120	0	0	0	0	1120	0	0	0	0
Lenalidomide	231 028	243 516	243 516	243 516	243 516	231 028	243 516	243 516	243 516	243 516
Dexamethasone	270	234	234	234	234	216	117	117	117	117
Total regimen DAC	433 286	381 186	381 186	381 186	381 186	421 196	371 769	331 305	331 305	331 305
Total treatment cost (regimen DAC + drug administration cost), $	436 856	382 096	382 096	382 096	382 096	429 700	375 943	334 161	334 161	334 161
Cumulative total cost of treatment, $	435 391	816 460	1 197 529	1 578 598	1 959 667	429 628	805 571	1 139 732	1 473 893	1 808 054
**Scenario 2: Median weight 66–75 kg, HOPA rounding guidance applied (one 500 mg vial and two 100 mg vials of isatuximab)**										
DAC of each drug per dosing schedule, $										
Anti-CD38[a]	200 868	137 436	137 436	137 436	137 436	165 228	112 119	76 713	76 713	76 713
Bortezomib SC	1120	0	0	0	0	1120	0	0	0	0
Lenalidomide	231 028	243 516	243 516	243 516	243 516	231 028	243 516	243 516	243 516	243 516
Dexamethasone	216	117	117	117	117	270	234	234	234	234
Total regimen DAC	433 223	381 069	381 069	381 069	381 069	397 592	355 752	320 346	320 346	320 346
Total treatment cost (regimen DAC + drug administration cost), $	436 856	382 096	382 096	382 096	382 096	406 096	359 926	323 202	323 202	323 202
Cumulative cost of treatment, $	436 856	818 952	1 201 048	1 583 144	1 965 240	406 096	766 022	1 089 224	1 412 426	1 735 628
**Scenario 3: Median weight 86-95 kg, HOPA rounding guidance applied (one 500 mg vial and four 100 mg vials of isatuximab)**										
DAC of each drug per dosing schedule, $										
Anti-CD38[a]	200 868	137 436	137 436	137 436	137 436	212 436	144 153	98 631	98 631	98 631
Bortezomib SC	1120	0	0	0	0	1120	0	0	0	0

Table 1. Number of Doses, DAC, and Administration Cost of DVRd and IsaVRd Across Weight Ranges 66-75 kg, 76-85 kg, and 86-95 kg

	DVRd					IsaVRd				
	**Year 1**	**Year 2**	**Year 3**	**Year 4**	**Year 5**	**Year 1**	**Year 2**	**Year 3**	**Year 4**	**Year 5**
Lenalidomide	231 028	243 516	243 516	243 516	243 516	231 028	243 516	243 516	243 516	243 516
Dexamethasone	270	234	234	234	234	216	117	117	117	117
Total regimen DAC	433 286	381 186	381 186	381 186	381 186	444 800	387 786	342 264	342 264	342 264
Total treatment cost (regimen DAC + drug administration cost), $	436 394	381 877	381 877	381 877	381 877	452 818	391 799	345 010	345 010	345 010
Cumulative cost of treatment, $	435 391	381 069	381 069	381 069	381 069	453 232	845 192	1 190 312	1 535 432	1 880 552

Abbreviations: DAC, drug acquisition costs; DVRd,daratumumab in combination with bortezomib, lenalidomide, and dexamethasone; IsaVRd, isatuximab in combination with bortezomib, lenalidomide, and dexamethasone; mAb, monoclonal antibody.

[a]All figures were plotted using cost of dexamethasone for patients age <75 years.

[b]The brand products used for this analysis were Darzalex Faspro® (daratumumab SC; Janssen Biotech, Inc.) and Sarclisa® (isatuximab; Sanofi-Aventis U.S., LLC).

[c]Same DAC and drug administration cost as the values used in Gupta-Werner et al was used in this calcuation: daratumumab SC, $10572; isatuximab, $843 for 100 mg and $4215 for 500 mg vials. Administration costs for procedure were assumed the same as the values used in Table 2 in Gupta-Werner et al.[1]

Figure 1. Effect of Variable Assumptions on a Comprehensive Economic Analysis on Drug Acquisition Cost, Administration Cost, and Total Treatment Cost of DVRd and IsaVRd[a,b,c]

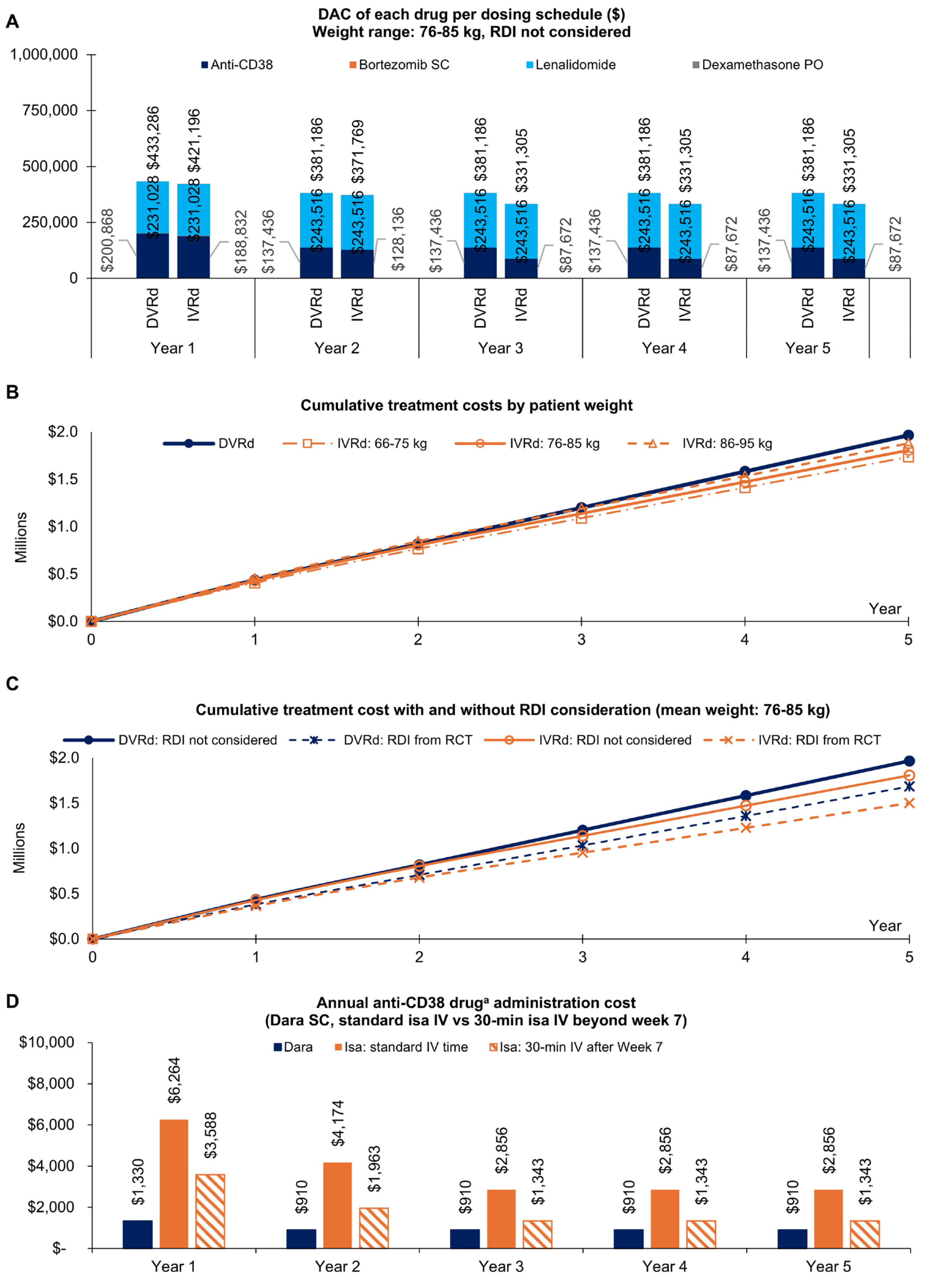

(**A**) Annual DAC of DVRd and IsaVRd regimens for patient weighting 76-85 kg; (**B**) cumulative treatment costs of DVRd and IsaVRd across patient weight ranges of 66-75 kg, 76-85 kg, and 86-95 kg; (**C**) impact of relative dose intensity on DAC; (**D**) administration costs for daratumumab SC, isatuximab standard IV, and isatuximab 30-minute IV.

Abbreviations: DAC, drug acquisition costs; DVRd, daratumumab in combination with bortezomib, lenalidomide, and dexamethasone; IsaVRd, isatuximab in combination with bortezomib, lenalidomide, and dexamethasone; mAb, monoclonal antibody.

[a]All figures were plotted using cost of dexamethasone for patients age <75.

[b]The brand products used for this analysis were Darzalex Faspro® (daratumumab SC; Janssen Biotech, Inc.) and Sarclisa® (isatuximab; Sanofi-Aventis U.S., LLC).

[c]Same DAC and drug administration cost as the values used in Gupta-Werner et al. was used in this calcuation: daratumumab SC, $10572; isatuximab, $843 for 100 mg and $4215 for 500 mg vials. Administration costs for procedure were assumed the same as the values used in Table 2 in Grupta-Werner et al.[1]
